# Noise Pollution: EU Ramps Up Road Improvements

**Published:** 2004-08

**Authors:** David C. Holzman

Noise costs the European Union (EU) 10–40 billion annually, by various estimates, with roughly half of this due to road noise. Contributing factors include medical costs, reduced worker productivity, and *de facto* condemnation of noise-exposed land. Due mostly to the demands of greater population density, European noise mitigation efforts are far ahead of those of the United States, and U.S. officials are paying attention: this spring, officials and researchers toured the best European projects.

Tires hitting pavement can cause as much as 90% of road traffic noise, depending on the traffic conditions, vehicle type, and driving style, says Ulf Sandberg, a senior research scientist at the Swedish National Road and Transport Research Institute. The treads squeeze air as they strike the road and snap as they pull away, which sets the tread and sidewalls vibrating. The upward-curving treads and the road surface form a “horn,” amplifying the cacophony, and the highway surface reflects the noise, says Roger L. Wayson, an associate professor of civil and environmental engineering at the University of Central Florida.

One strategy to fight road/tire noise is single-layer porous highways sitting on an asphalt concrete foundation, which cover hundreds of miles in Europe. The pores, created by using stones of similar size in the asphalt mix, are thought to dampen the hiss of the pumped air and to impair the acoustic reflectivity of the road surface. The resulting 3-decibel reduction over a conventional European highway is readily perceptible.

Besides dampening noise, porous surfaces drain rain, potentially reducing accidents. But they also drain winter road salt (sand can’t be used because it blocks the pores). Europeans use wetted salt, which sticks longer to the road, but U.S. observers worry that more salt use could trade one environmental problem for another. Still, single-layer porous surfaces are successful in the European countries visited, says Christopher Corbisier, a civil engineer and noise specialist with the Federal Highway Administration who took the recent tour.

More experimental are roadways with two layers of porous asphalt atop the foundation, which shave another several decibels from the din, says Gijsjan van Blokland, general manager of the Dutch company M&P Consulting Engineers. At short test sites in Italy and the Netherlands noise-absorbing Helmholtz resonators are embedded in the concrete foundation, cutting several decibels more. These highways tend to wear more quickly than the single-layered ones.

Although noise costs have not been estimated for the United States, road noise is still a concern here. In the United States, noise mitigation must be considered if residential exposures reach 66 decibels, although it is not required if deemed not reasonably feasible. Tall concrete noise barriers are typically used, but cost more than $1 million per mile. Quiet roads offer a potentially cheaper, more aesthetically pleasing alternative.

